# Treatment outcome in a specialized unit for adults with severe and extreme anorexia nervosa at one-year follow up

**DOI:** 10.1186/s40337-025-01374-z

**Published:** 2025-08-25

**Authors:** Adrian Meule, Eva P. Wuttke, Thorsten Koerner, Ulrich Cuntz, Ulrich Voderholzer

**Affiliations:** 1 Department of Psychology, University of Regensburg, Universitätsstraße 31, 93053 Regensburg, Germany; 2 Department of Psychiatry and Psychotherapy, LMU University Hospital, LMU Munich, Munich, Germany; 3 Schoen Clinic Roseneck, Prien am Chiemsee, Germany; 4 Forschungsprogramm für Psychotherapieevaluation im komplexen Therapiesetting, Paracelsus Medical University, Salzburg, Austria; 5 Department of Psychiatry and Psychotherapy, University Hospital of Freiburg, Freiburg, Germany

**Keywords:** Inpatient treatment, Severe and enduring anorexia nervosa, Body mass index, Eating disorders, Psychotherapy

## Abstract

**Background:**

Inpatient treatment successfully increases body weight and decreases eating disorder and associated symptoms in patients with anorexia nervosa (AN). However, relapse rates are high, particularly within the first year after discharge.

**Methods:**

We examined treatment outcome one year after discharge in adults with AN (*N* = 80, 2 males; BMI at admission: *M* = 13.2 kg/m^2^, *SD* = 1.79) who received treatment in a specialized inpatient unit for AN patients with severe underweight (body mass index < 15 kg/m^2^) and/or excessive purging or exercising.

**Results:**

From admission to discharge, body weight and self-reported life satisfaction significantly increased and self-reported eating disorder symptoms, depressive symptoms, and compulsive exercise significantly decreased. From discharge to follow up, life satisfaction and body weight decreased, and eating disorder symptoms, depressive symptoms, and compulsive exercise increased, although 87% of patients indicated to have received some kind of eating disorder treatment in the past six months. At follow up, the majority of patients indicated that they regularly ate three meals per day in the past week, including consumption of high-calorie, formerly forbidden foods. However, only a minority of patients indicated that they adhered to the hospital’s guidelines on portion sizes. Patients’ self-reported desired body weight at follow up was significantly higher than their current body weight.

**Conclusions:**

While inpatient treatment results in substantial improvements that are partially maintained after discharge, severe and extreme cases of AN require more long-lasting, alternating treatment approaches (e.g., interval treatment) to ensure long-term recovery.

## Abstract (plain language summary)

This study examined outcomes of inpatient treatment for adults with anorexia nervosa one year after discharge. Initially, patients showed significant improvements in body weight, life satisfaction, and reductions in eating disorder symptoms, depression, and compulsive exercise. However, within a year after leaving the hospital, many patients experienced a decline in life satisfaction and body weight, along with a resurgence of eating disorder symptoms, depression, and compulsive exercise, despite most receiving some form of treatment during this period. While most patients reported eating three meals a day and consuming high-calorie foods, few followed the hospital's portion size guidelines. It is concluded that while inpatient treatment is effective, severe cases of anorexia nervosa need ongoing, innovative treatment approaches to support long-term recovery.

## Background

Anorexia nervosa (AN) is an eating disorder that is characterized by significantly low body weight, which is maintained by behaviors such as restrictive eating, purging (e.g., self-induced vomiting, misuse of laxatives), and excessive exercise, and typically associated with a fear of weight gain [2]. AN severity is assessed according to a person’s body mass index (BMI) in the current version of both the Diagnostic and Statistical Manual of Mental Disorders (DSM–5; [2]) and the International Classification of Diseases (ICD–11; [46]). In the DSM–5, severity is categorized as mild (BMI ≥ 17.0 kg/m^2^), moderate (BMI ≥ 16.0 kg/m^2^ and < 17.0 kg/m^2^), severe (BMI ≥ 15.0 kg/m^2^ and < 16.0 kg/m^2^), and extreme (BMI < 15.0 kg/m^2^). In the ICD–11, severity is categorized as AN in recovery with normal body weight (BMI ≥ 18.5 kg/m^2^), AN with significantly low body weight (BMI < 18.5 kg/m^2^ and ≥ 14 kg/m^2^), and AN with dangerously low body weight (BMI < 14 kg/m^2^).

There have also been suggestions to alternatively consider overvaluation of weight and shape, illness duration, and previous treatments as indicators for AN severity [15, 45]. For example, some studies defined so-called severe and enduring AN based on an illness duration of at least seven years [8]. Hay and Touyz [29] proposed to define severe and enduring AN as (1) a persistent state of dietary restriction, underweight, and overvaluation of weight and shape with functional impairment, (2) illness duration of longer than three years, and (3) exposure to at least two evidence-based treatments.

Having severe and extreme AN is usually an indication for inpatient treatment, which often includes involuntary admission and enteral nutrition [1, 27]. However, voluntary inpatient treatment that includes a high-calorie refeeding schedule with supervised meals, individual psychotherapy sessions, group therapies, close medical monitoring, and micronutrient supplementation also successfully improves health status in persons with extreme AN [12, 32].

Risk of relapse is high in persons with AN, particularly in the first year after inpatient treatment [7]. Approximately 25–35% of AN patients need to be rehospitalized in that period [16, 26, 38]. However, most studies report that body weight at discharge is—on average—largely maintained at one-year follow up in both adolescents and adults, the majority of which received psychotherapeutic aftercare [13, 16, 26, 38, 42]. Studies that examined outcome after inpatient treatment in persons with severe and extreme AN are rare. For example, Abry et al. [1] recently reported on a study that compared involuntarily and voluntarily treated AN patients with an average BMI of 12 kg/m^2^ at admission, in which BMI increased from discharge to follow up. However, these findings may be biased by small sample size and high attrition rates.

The aim of the current study was to examine treatment outcome one year after discharge in a sample of persons with AN (94% being categorized as having severe or extreme AN according to DSM–5, see below) treated at a specialized inpatient unit. Specifically, we examined changes in BMI, eating disorder psychopathology, compulsive exercise, depressive symptoms, and life satisfaction across admission, discharge, and one-year follow up. We also tested whether BMI at admission, classifying patients as having severe and enduring AN, and illness duration would moderate changes over time of these variables. Finally, we also report on eating-, weight-, and treatment-related questions at follow up.

## Methods

### Sample characteristics

**Fig. 1:**
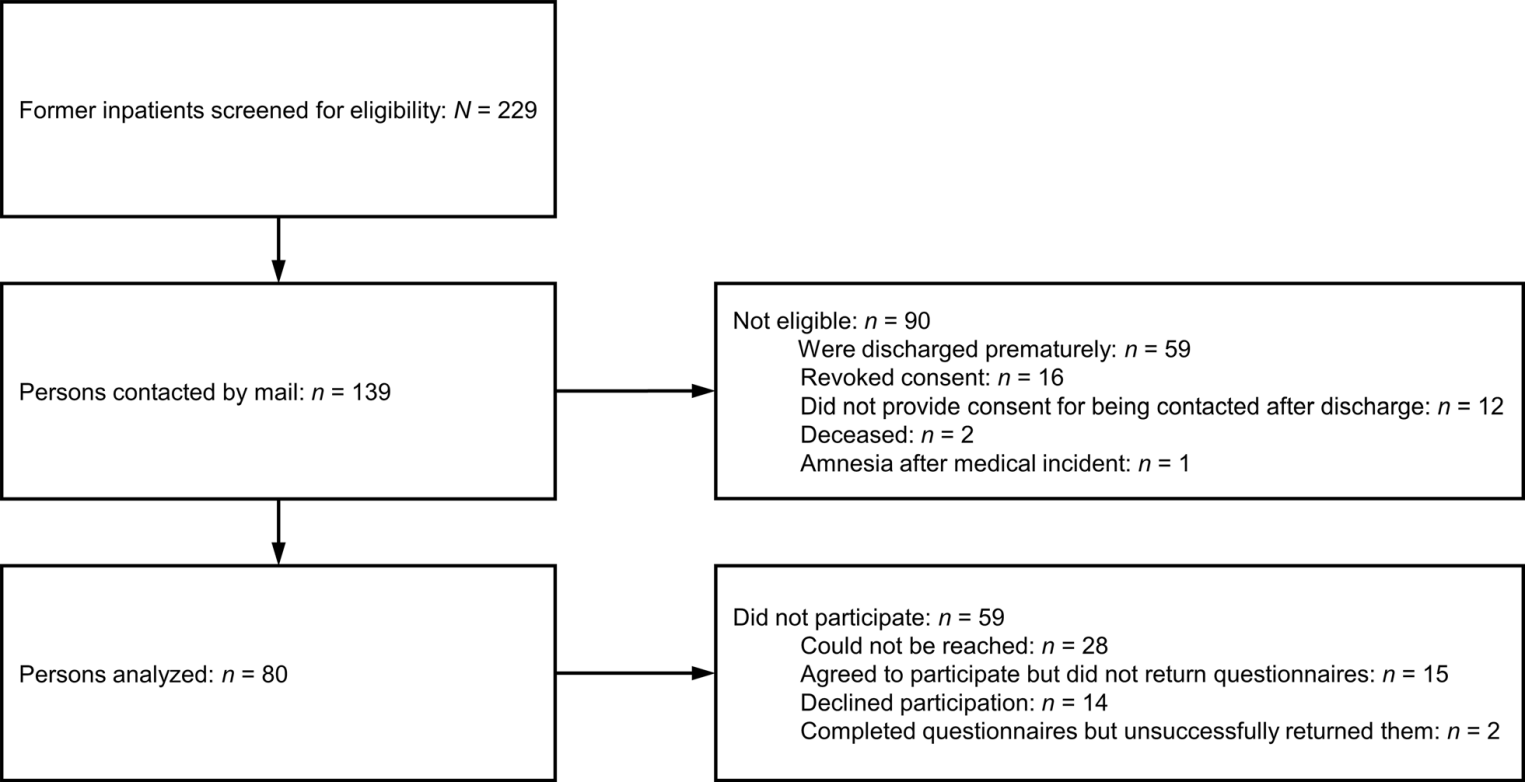
Participant flow

The study was approved by the ethics committee of the University Hospital of the LMU Munich. One-hundred and thirty-nine adults with AN who received inpatient treatment at the Schoen Clinic Roseneck (Rosenheim, Germany) between 2018 and 2020 were contacted one year after discharge between 2019 and 2021 (Fig. 1). Of these, 80 persons participated in the study (98% female, *n* = 78; mean age: *M* = 27.3 years, *SD* = 10.2). Fifty-three persons (66%) had restrictive-type AN (ICD–10 code F50.00), 26 (33%) had binge/purge-type AN (ICD–10 code F50.01), and one (1%) had atypical AN (ICD–10 code F50.1). Forty persons (50%) had at least one comorbid mental disorder, the most common of which were anxiety disorders (ICD–10 code F4; *n* = 26, 33%) and affective disorders (ICD–10 code F3; *n* = 17, 21%). A subset of patients received antidepressant (*n*/*N* = 13/53, 25%) or antipsychotic (*n*/*N* = 15/53, 28%) medication during the stay (information not available for 27 patients).

When using the severity classification according to ICD–11 [46], 66.3% (*n* = 53) had AN with dangerously low body weight (BMI < 14.0 kg/m^2^) and 33.8% (*n* = 27) had AN with significantly low body weight (BMI ≥ 14.0 kg/m^2^ and < 18.5 kg/m^2^). When using the severity classification according to DSM–5 [2], 85.0% (*n* = 68) had extreme AN (BMI < 15.0 kg/m^2^), 8.8% (*n* = 7) had severe AN (BMI ≥ 15.0 kg/m^2^ and < 16.0 kg/m^2^), 3.8% (*n* = 3) had moderate AN (BMI ≥ 16.0 kg/m^2^ and < 17.0 kg/m^2^), and 2.5% (*n* = 2) had mild AN (BMI ≥ 17.0 kg/m^2^). Mean illness duration was 10.1 years (*SD* = 9.74, Range: 1–40) and 55.0% (*n* = 44) had an illness duration of at least seven years and 45.0% (*n* = 36) had an illness duration of less than seven years. Illness duration did not relate to BMI at admission (*r*_pb_ = 0.05, *p* = 0.680). The mean number of previous treatments was 3.4 (*SD* = 3.7, Range 0–16). When using the criteria by Hay and Touyz [29], 53.3% (*n* = 41) had severe and enduring AN and 46.8% (*n* = 36) did not meet the criteria for severe and enduring AN (information not available for three patients).

All patients were treated in a specialized inpatient unit for AN patients with severe underweight (BMI < 15 kg/m^2^) and/or excessive purging or exercising (length of stay: *M* = 102.7 days, *SD* = 52.2). The treatment at the hospital adheres to the German S3-guidelines for the treatment of AN in terms of admission criteria, treatment elements, and therapy goals [3]. Thus, patients received a cognitive-behavioral therapy-oriented, multimodal AN treatment that included several treatment elements such as individual psychotherapy sessions, group therapy sessions, exercise therapy, meal preparation classes, body image exposure, nutrition counseling, and food intake protocols as well as clinical management of medical complications, including micronutrient supplementation. The treatment includes a high-calorie refeeding schedule (starting on the first day of treatment) that aims at a weight gain of 0.7–1.0 kg per week for all underweight AN patients. This schedule includes three meals per day, each having approximately 700 kcal and, thus, totaling to a daily caloric intake of approximately 2100 kcal. Meals are supervised by a staff member (nurse, psychotherapist, or physician) in earlier treatment stages. The schedule is individually tailored if patients do not finish their meals or do not show the expected weight gain by increasing portion size, adding snacks between meals, or offering sip feeds. As normalization of eating behavior is one of the therapeutic goals, patients do not receive nasogastric feeding. Patients can choose between vegetarian and non-vegetarian menus; vegan menus are not offered. Compared to other eating disorder units at the hospital, the treatment at the specialized unit is more intensive and structured, with enhanced medical monitoring, closer behavioral supervision (e.g., during meals and to ensure limiting physical activity and preventing purging behaviors), daily weighing, and more frequent therapeutic contact tailored to patients with severe AN.

### Measures

*BMI*. Body weight and height were measured at the hospital at admission and discharge and participants self-reported their current height and weight at follow up. BMI was calculated as kg/m^2^.

*Eating Disorder Examination–Questionnaire (EDE–Q)*. Eating disorder symptomatology was assessed with the German version [30] of the EDE–Q [22]. The EDE–Q has 28 items, six of which assess the frequency of binge and purge behaviors in the past 28 days and are not included in the total score. The other 22 items are answered on a seven-point scale (0–6) with different response labels. Higher mean total scores indicate higher eating disorder symptomatology. Internal reliability at admission, discharge, and follow up ranged between ω = 0.95 and 0.98.

*Commitment to Exercise Scale (CES).* Compulsive exercise was assessed with the German version [47] of the CES [17]. The CES has eight items and, in the original version, these were answered on a visual analogue scale with different anchors. In the current study, we applied a four-point scale (e.g., 1 = *never* to 4 = *always*) response format, in line with other studies [19, 20, 44]. Higher mean total scores indicate higher compulsive exercising. Internal reliability at admission, discharge, and follow up ranged between ω = 0.95 and 0.97.

*Beck Depression Inventory–Revised (BDI–II)*. Depressive symptoms were assessed with the German version [28] of the BDI–II [5]. The BDI–II has 21 items that are answered on a four-point scale (0–3) with different response labels. Higher total sum scores indicate higher depressive symptomatology. Internal reliability at admission, discharge, and follow up ranged between ω = 0.91 and 0.95.

*Satisfaction With Life Scale (SWLS)*. Life satisfaction was assessed with the German version [25] of the SWLS [18]. The SWLS has five items that are answered on a seven-point scale (1 = *strongly disagree* to 7 = *strongly agree*). Higher total sum scores indicate higher life satisfaction. Internal reliability at admission, discharge, and follow up ranged between ω = 0.88 and 0.92.

*Eating- and weight-related questions at follow up.* Current meal frequency was assessed with three questions asking “On how many days in the past seven days have you had breakfast/lunch/dinner?”. Responses were recorded on an eight-point scale from 0 to 7 (ω = 0.77) and averaged to a mean score. Current meal portions were assessed with three questions asking “On how many days in the past seven days have you eaten according to the portion size guidelines by the hospital at breakfast/lunch/dinner?”. Responses were recorded on an eight-point scale from 0 to 7 (ω = 0.86) and averaged to a mean score. Current intake of high-calorie foods was assessed with one question asking “On how many days in the past seven days have you eaten high-caloric (previously “forbidden”) foods?”. Responses were recorded on an eight-point scale from 0 to 7. Current desired body weight was assessed with one item stating “My current desired body weight is … kg.”.

*Questions on current and past treatments at follow up*. Eating disorder treatments were assessed with five questions asking “Have you received outpatient [physician]/outpatient [psychotherapist]/outpatient [group]/daypatient/inpatient eating disorder treatment in the past six months?” (yes/no). Current psychotherapeutic treatment was assessed with one question asking “Do you currently receive psychotherapeutic treatment?” (yes/no). Current psychopharmacological treatment was assessed with one question asking “Do you currently take medication to treat your mental disorder?” (yes/no, if yes enter name of drugs).

### Data analyses

Statistical analyses were conducted with R version 4.5.0 (https://www.r-project.org) in RStudio version 2025.05.0 (https://posit.co). As assumptions of the general linear model are often violated when analyzing clinical psychology data, it has been suggested preferring nonparametric and robust analysis techniques [23]. Therefore, changes in BMI as well as EDE–Q, CES, BDI–II, and SWLS scores across admission, discharge, and follow up were examined with robust mixed models with the *robustlmm* package [33]. Specifically, separate models were calculated with either BMI, EDE–Q, CES, BDI–II, or SWLS scores as dependent variable. Fixed effects of time were added by including first- and second-order orthogonal polynomials of the time term as independent variables, modeling linear and non-linear changes across the three measurements [40]. The models also included a random intercept (i.e., person-level random variability in scores at admission). As the *robustlmm* package does not produce parameter-specific *p*-values, we used the workaround described by Geniole et al. [24]. Specifically, non-robust models were fitted with the *lme4* package [4] to obtain Satterthwaite-approximated degrees of freedom and two-sided *p*-values were then computed for the robust *t*-values using the approximated degrees of freedom. Pairwise comparisons (admission vs. discharge, discharge vs. follow up) were computed with the *emmeans* package [34] and Cohen’s *d* was computed as effect size with the *effectsize* package [6]. Note that—although there were missing data for each dependent variable—these mixed models included all 80 cases because of the restricted maximum likelihood estimation.

In another set of robust mixed models, we added the fixed effect of BMI at admission and its interaction with the two time terms to examine whether changes in BMI and questionnaire scores across admission, discharge, and follow up would be moderated by BMI at admission. Specifically, in contrast to the models described above that only included *time* and *time*^*2*^ as independent variables, these models included *time*, *time*^*2*^, *BMI at admission*, *time* × *BMI at admission*, and *time*^*2*^ × *BMI at admission* as independent variables. Note, however, that the only effect of interest here was the interaction effect *time*^*2*^ × *BMI at admission*, testing whether the non-linear changes across time would differ as a function of BMI at admission but all “subordinate” effects have to be included in such a model as well. Similar models were run to examine whether changes would be moderated by severe and enduring AN groups and illness duration.

Internal reliability coefficients (McDonald’s ω) for all questionnaires for which items were averaged or summed were obtained with the *psych* package [43]. Descriptive statistics for variables used for sample description, eating- and weight-related questions at follow up, and questions on current and past treatments at follow up were obtained with the *summarytools* package [11]. Meal frequency and meal portions as well as current and desired body weight at follow up were compared with Wilcoxon paired signed-rank tests with the *rcompanion* package [35]. The data and code with which all analyses can be reproduced are available at https://osf.io/3uypd.

## Results

### BMI

**Fig. 2:**
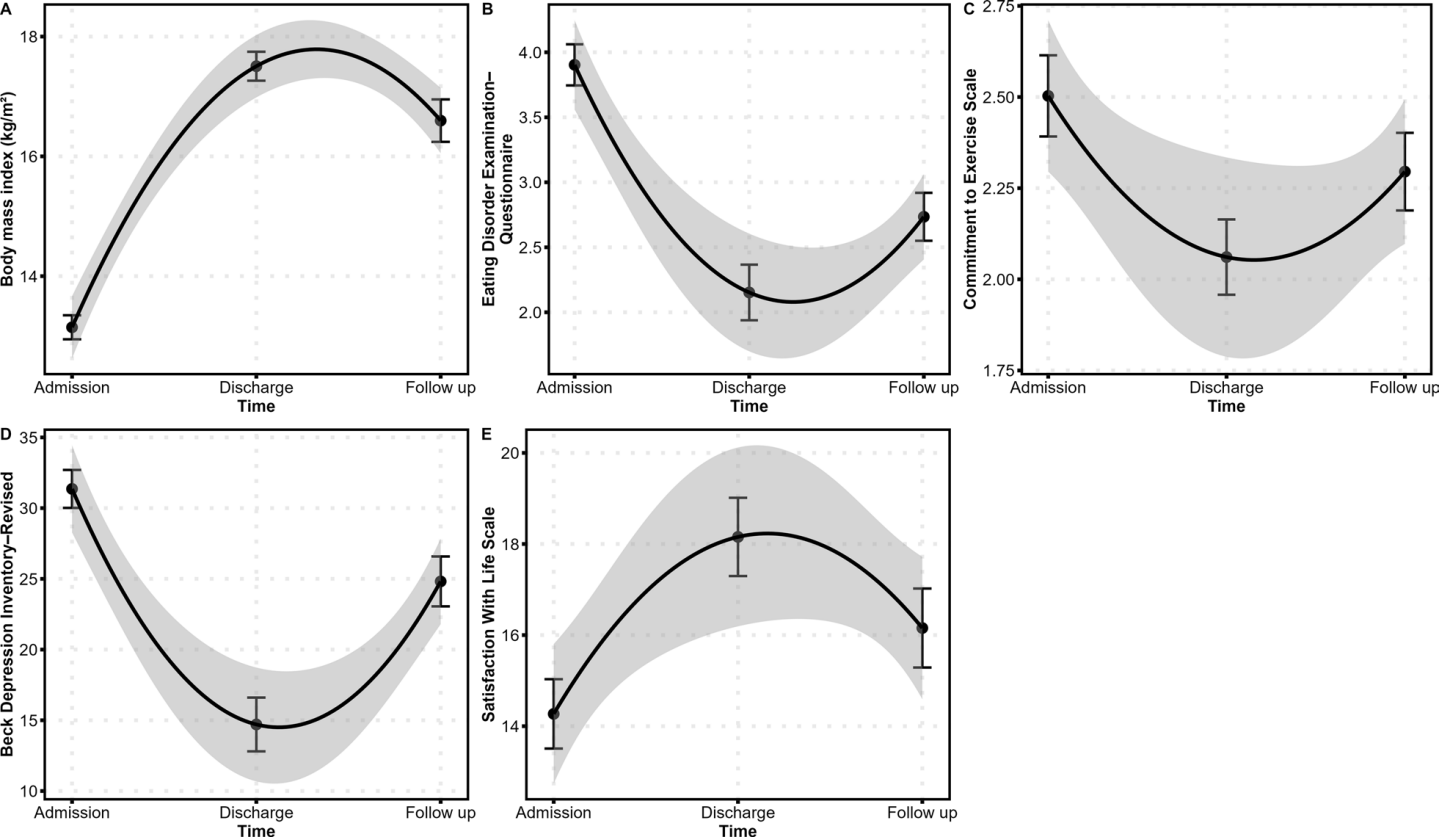
Mean **A** body mass index and scores on the **B** Eating Disorder Examination–Questionnaire, **C** Commitment to Exercise Scale, **D** Beck Depression Inventory–Revised, and **E** Satisfaction With Life Scale at admission, discharge, and follow up. Error bars indicate standard error of the mean. The black lines are second-order polynomial fit lines indicating non-linear change over time and the grey-shaded areas represent 95% confidence intervals. Note that numbers in the different panels are not comparable because of different scaling and scoring of variables

**Fig. 3:**
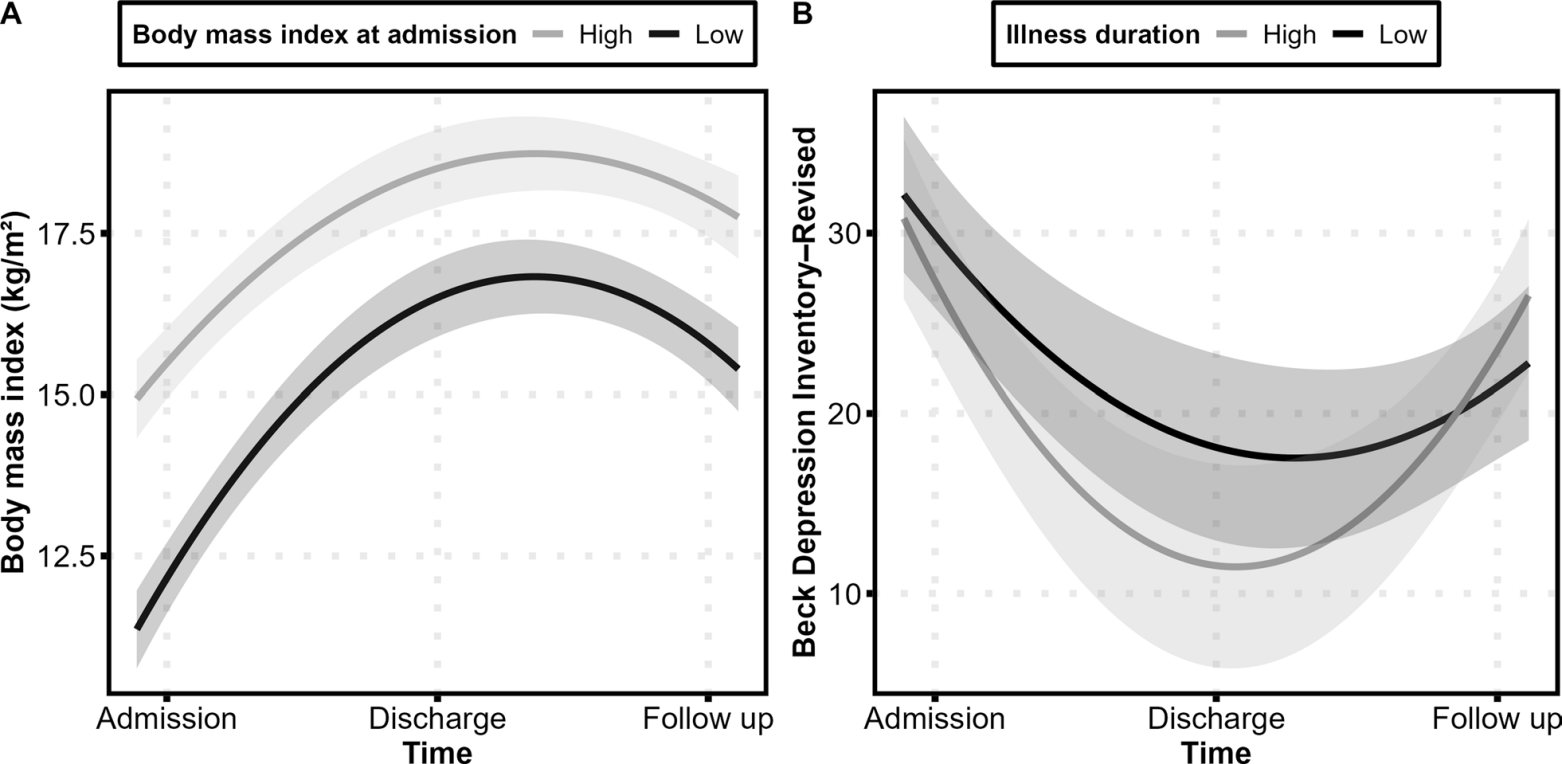
Changes across admission, discharge, and follow up in **A** body mass index (BMI) as a function of BMI at admission and **B** scores on the Beck Depression Inventory–Revised as a function of illness duration. The lines are second-order polynomial fit lines indicating non-linear change over time and the grey-shaded areas represent 95% confidence intervals. Note that *high* and *low* refer to ± 1 *SD* from the sample’s mean BMI at admission and illness duration, respectively. These values are arbitrarily chosen for visualizing the interaction effect time^2^ × BMI at admission and time^2^ × illness duration in the robust mixed models. That is, BMI at admission and illness duration were used as continuous variables in these analyses and not categorized into groups

BMI changed non-linearly across the three measurements (effect of *time*^*2*^: *b* =  − 2.27, *SE* = 0.18, *p* < 0.001). Specifically, BMI increased from admission to discharge (*b* = 4.38, *SE* = 0.25, *p* < 0.001, *d* = 2.16) and decreased from discharge to follow up (*b* =  − 1.17, *SE* = 0.26, *p* < 0.001, *d* =  − 0.37; Fig. 2A). Changes in BMI differed as a function of BMI at admission (interaction effect of *time*^*2*^ × *BMI at admission*: *b* = 0.23, *SE* = 0.09, *p* = 0.010): lower BMI at admission related to steeper increases in BMI across measurements (Fig. 3A). Changes in BMI did not differ as a function of severe and enduring AN groups (interaction effect of *time*^*2*^ × *groups*: *b* = 0.09, *SE* = 0.37, *p* = 0.817) or illness duration (interaction effect of *time*^*2*^ × *illness duration*: *b* = 0.003, *SE* = 0.02, *p* = 0.861).

### EDE–Q

Eating disorder symptomatology changed non-linearly across the three measurements (effect of *time*^*2*^: *b* = 0.93, *SE* = 0.16, *p* < 0.001). Specifically, EDE–Q scores decreased from admission to discharge (*b* =  − 1.69, *SE* = 0.21, *p* < 0.001, *d* =  − 1.36) and increased from discharge to follow up (*b* = 0.60, *SE* = 0.21, *p* = 0.004, *d* = 0.36; Fig. 2B). Changes in EDE–Q scores did not differ as a function of BMI at admission (interaction effect of *time*^*2*^ × *BMI at admission*: *b* = 0.05, *SE* = 0.09, *p* = 0.579) or severe and enduring AN groups (interaction effect of *time*^*2*^ × *groups*: *b* = 0.01, *SE* = 0.32, *p* = 0.969) or illness duration (interaction effect of *time*^*2*^ × *illness duration*: *b* = 0.02, *SE* = 0.02, *p* = 0.247).

### CES

Compulsive exercise changed non-linearly across the three measurements (effect of *time*^*2*^: *b* = 0.30, *SE* = 0.08, *p* < 0.001). Specifically, CES scores decreased from admission to discharge (*b* =  − 0.44, *SE* = 0.11, *p* < 0.001, *d* =  − 0.69) and increased from discharge to follow up (*b* = 0.29, *SE* = 0.11, *p* = 0.007, *d* = 0.30; Fig. 2C). Changes in CES scores did not differ as a function of BMI at admission (interaction effect of *time*^*2*^ × *BMI at admission*: *b* = 0.03, *SE* = 0.05, *p* = 0.532) or severe and enduring AN groups (interaction effect of *time*^*2*^ × *groups*: *b* = 0.02, *SE* = 0.16, *p* = 0.889) or illness duration (interaction effect of *time*^*2*^ × *illness duration*: *b* = 0.003, *SE* = 0.01, *p* = 0.677).

### BDI–II

Depressive symptomatology changed non-linearly across the three measurements (effect of *time*^*2*^: *b* = 10.8, *SE* = 1.58, *p* < 0.001). Specifically, BDI–II scores decreased from admission to discharge (*b* =  − 17.1, *SE* = 2.13, *p* < 0.001, *d* =  − 1.27) and increased from discharge to follow up (*b* = 9.41, *SE* = 2.12, *p* < 0.001, *d* = 0.64; Fig. 2D). Changes in BDI–II scores did not differ as a function of BMI at admission (interaction effect of *time*^*2*^ × *BMI at admission*: *b* =  − 0.62, *SE* = 0.92, *p* = 0.503) or severe and enduring AN groups (interaction effect of *time*^*2*^ × *groups*: *b* = 3.17, *SE* = 3.33, *p* = 0.343). However, they differed as a function of illness duration (interaction effect of *time*^*2*^ × *illness duration*: *b* = 0.35, *SE* = 0.16, *p* = 0.028): a longer illness duration related to steeper decreases in BDI–II scores from admission to discharge and steeper increases in BDI–II scores from discharge to follow up (Fig. 3B).

### SWLS

Life satisfaction changed non-linearly across the three measurements (effect of *time*^*2*^: *b* =  − 2.23, *SE* = 0.69, *p* = 0.002). Specifically, SWLS scores increased from admission to discharge (*b* = 3.61, *SE* = 0.93, *p* < 0.001, *d* = 0.57) and decreased from discharge to follow up (*b* =  − 1.86, *SE* = 0.94, *p* = 0.048, *d* =  − 0.16; Fig. 2E). Changes in SWLS scores did not differ as a function of BMI at admission (interaction effect of *time*^*2*^ × *BMI at admission*: *b* = 0.11, *SE* = 0.41, *p* = 0.785) or severe and enduring AN groups (interaction effect of *time*^*2*^ × *groups*: *b* =  − 0.09, *SE* = 1.41, *p* = 0.952) or illness duration (interaction effect of *time*^*2*^ × *illness duration*: *b* =  − 0.06, *SE* = 0.07, *p* = 0.392).

### Eating- and weight-related questions at follow up

On average, participants reported to eat three meals per day on about six days in the past week (*M* = 6.14 days, *SD* = 1.52) but reported adhering to portion size guidelines on significantly fewer days (*M* = 3.77, *SD* = 2.59; *V* = 1649, *p* < 0.001, *r*_rb_ = 0.995). The majority of participants (90%, *n*/*N* = 71/79, information not available for one participant) indicated that they ate high-caloric, formerly forbidden foods on at least one day in the past week. Current self-reported body weight (*M* = 45.5 kg, *SD* = 9.16) was significantly lower than desired body weight (*M* = 47.6, *SD* = 8.93; *V* = 471, *p* = 0.002, *r*_rb_ = 0.468). In fact, 69% of participants (*n*/*N* = 43/62, information not available for 18 participants) indicated that their desired weight was higher than their current weight, 26% (*n*/*N* = 16/62) indicated that their desired weight was lower than their current weight, and 5% (*n*/*N* = 3/62) indicated that their desired weight was equal to their current weight. When converting desired weight into BMI (*M* = 17.5 kg/m^2^, *SD* = 2.79), the majority of participants (61%, *n*/*N* = 39/62) indicated that their desired BMI was lower than 18.5 kg/m^2^ (that is, in the underweight range according to the classification of the World Health Organization).

### Questions on current and past treatments at follow up

The majority of participants (87%, *n*/*N* = 68/78, information not available for two participants) indicated they received any eating disorder treatment in the past six months (outpatient [physician]: 42%, *n*/*N* = 33/78; outpatient [psychotherapist]: 63%, *n*/*N* = 49/78; outpatient [group]: 9%, *n*/*N* = 7/78; daypatient: 5%, *n*/*N* = 4/78; inpatient: 35%, *n*/*N* = 27/78) and that they currently received psychotherapeutic treatment (63%, *n*/*N* = 49/78). The minority of participants (44%, *n*/*N* = 35/79, information not available for one participant) indicated that they currently received psychopharmacological treatment (antidepressants: 35%, *n*/*N* = 28/79; antipsychotics: 19%, *n*/*N* = 15/79).

## Discussion

The current study examined treatment outcome in persons with AN treated at a specialized unit for severe and extreme AN across admission, discharge, and one-year follow up. Disorder-specific and related symptoms substantially improved during treatment but somewhat declined after treatment. For example, average BMI increased from 13.2 kg/m^2^ at admission (with all patients being underweight [BMI < 18.5 kg/m^2^]) to 17.5 kg/m^2^ at discharge (with 27 of 80 patients [33.8%] having a BMI ≥ 18.5 kg/m^2^) but decreased to 16.6 kg/m^2^ after one year (with 18 of 73 patients [22.5%] having a BMI ≥ 18.5 kg/m^2^). These changes were moderated by BMI at admission such that a lower BMI at admission related to steeper weight gain from admission to discharge. This effect might be explained by several factors. Biologically, larger weight gain in those with lower BMI may be due to lower resting metabolic rate at the beginning of treatment. Psychologically, many patients accept that they have to gain weight during treatment but refuse to exceed certain self-imposed thresholds (e.g., 50 kg), which are reached faster by those who start with higher BMI at admission. Besides these explanations, however, it has been observed that—due to regression to the mean—there is always a correlation between baseline scores and change scores, regardless of any treatment effects [10]. Thus, while it appears that AN patients with a lower BMI at admission achieve a higher weight gain during inpatient treatment, an incorrect interpretation would be to conclude that the treatment is more effective for these patients [37].

Although most patients had severe and extreme AN according to their BMI at admission, only about half of the sample met the criteria for severe and enduring AN by Hay and Touyz [29]. Furthermore, changes in AN symptomatology during and after treatment did not differ between those classified as having severe and enduring AN and those not classified as having severe and enduring AN. Moreover, patients with a longer illness duration even showed stronger decreases in depressive symptoms from admission to discharge, which yet were not maintained at follow up. Thus, the current findings do not indicate that patients with severe and enduring AN have a less favorable treatment outcome or should be treated differently than patients without severe and enduring AN, as has been discussed in the literature [45].

Accompanying the pattern of changes in body weight, eating disorder psychopathology, compulsive exercise, and depressive symptoms decreased during inpatient treatment but increased afterwards. Similarly, life satisfaction increased during inpatient treatment but decreased afterwards. These changes were not moderated by BMI at admission, indicating that patients showed improvements in these aspects independent of their initial body weight. This finding dovetails with suggestions that the DSM–5 severity specifiers based on BMI may not reflect meaningful differences in terms of psychopathology, distress, and prognosis [14, 15]. Yet, the current results still suggest that persons with severe and extreme AN have a poorer prognosis than those with a higher body weight as studies that examined samples that also included (or were mainly composed of) persons with mild and moderate severity reported a more favorable treatment outcome at one-year follow up [13, 16, 26, 38, 42].

The finding that the treatment led to substantial symptom improvements but—despite that most patients received psychotherapeutic aftercare—there were still refractory symptoms after discharge is also reflected in the eating- and weight-related variables assessed at follow up. While the majority of participants indicated that they regularly ate three meals per day that also included high-caloric, formerly forbidden foods, most participants did not adhere to the portion size guidelines from the hospital. Furthermore, while participants’ desired body weight at follow up was higher than their current body weight—possibly indicating a high motivation to recover—this desired body weight was still in the underweight range for most participants.

Interpretation of results is limited to inpatients with AN treated in Germany and, thus, may not translate to other countries with different healthcare systems (e.g., length of stay is usually shorter in the USA than in Europe; [31]). Furthermore, body weight at follow up was based on self-report, which may be biased. Yet, persons with AN are extremely accurate when self-reporting their own weight. For example, self-reported weight has been found to be more accurate in women with AN than in normal-weight and overweight women [21]. Although it has been found that they slightly overestimate their weight, this overestimation is less than one kilogram on average [9, 36, 39]. Thus, it is unlikely that using self-report of current weight at follow up substantially affected results of the current study. Yet, other variables such as eating behaviors and compulsive exercise were also based on self-report and, thus, influences like recall bias or demand effects cannot be excluded. Therefore, including objective measures of these variables would be desirable in future studies.

In conclusion, the current study shows that voluntary inpatient treatment that includes a high-calorie refeeding schedule with supervised meals, individual psychotherapy sessions, group therapies, close medical monitoring, and micronutrient supplementation in patients with severe and extreme AN leads to substantial improvements that are partially maintained after discharge. However, symptom improvements somewhat deteriorate after discharge despite psychotherapeutic aftercare, indicating that persons with severe and extreme AN require more long-lasting, alternating treatment approaches (e.g., interval treatment; [41]) to ensure long-term recovery.

## Data Availability

The data and code with which all results can be reproduced can be accessed at https://osf.io/3uypd.
